# Combining ZHENG Theory and High-Throughput Expression Data to Predict New Effects of Chinese Herbal Formulae

**DOI:** 10.1155/2012/986427

**Published:** 2012-05-14

**Authors:** Shuhao Yu, Zhizhong Guo, Yan Guan, Yi-Yu Lu, Pei Hao, Yixue Li, Shi-Bing Su

**Affiliations:** ^1^College of Life Science and Biotechnology, Shanghai Jiaotong University, 800 Dongchuan Road, Shanghai 200240, China; ^2^Research Center for Complex System of Traditional Chinese Medicine, Shanghai University of Traditional Chinese Medicine, 1200 Cailun Road, Shanghai 201203, China; ^3^Key Lab of Systems Biology/Key Laboratory of Synthetic Biology, Shanghai Institutes for Biological Sciences, Chinese Academy of Sciences, 320 Yueyang Road, Shanghai 200031, China

## Abstract

ZHENG is the key theory in traditional Chinese medicine (TCM) and it is very important to find the molecular pharmacology of traditional Chinese herbal formulae. One ZHENG is related to many diseases and the herbal formulae are aiming to ZHENG. Therefore, many herbal formulae whose effects on a certain disease have been confirmed might also treat other diseases with the same ZHENG. In this study, the microarrays collected from patients with QiXuXueYu ZHENG (Qi-deficiency and Blood-stasis syndrome) before treatment and after being treated with Fuzheng Huayu Capsule were analyzed by a high-throughput gene microarrays-based drug similarity comparison method, which could find the small molecules which had similar effects with Fuzheng Huayu Capsule. Besides getting the results of anti-inflammatory and anti-fibrosis drugs which embody the known effect of Fuzheng Huayu Capsule, many other small molecules were screened out and could reflect other types of effects of this formula in treating QiXuXueYu ZHENG, including anti-hyperglycemic, anti-hyperlipidemic, hyposenstive effect. Then we integrated this information to display the effect of Fuzheng Huayu Capsule and its potential multiple-target molecular pharmacology. Moreover, through using clinical blood-tested data to verify our prediction, Fuzheng Huayu Capsule was proved to have effects on diabetes and dyslipidemia.

## 1. Introduction

The traditional Chinese medicine (TCM) ZHENG, also known as TCM syndrome, is the key theory in TCM and the important diagnostic principle for TCM therapy [1]. It is very important to describe ZHENG in molecular level or find the molecular marks in ZHENG identification or classification, and then find the molecular pharmacology of traditional Chinese herbal formulae whose treatment are based the ZHENG.

 Most current researches in ZHENG and herbal formulae were guided by the theory of western medicine, their study objects are “disease,” not “ZHENG.” So these researchers had got a certain “disease,” and did some ZHENG identification and ZHENG classification work based on that certain disease [2, 3], though using high-throughput gene microarrays. Similarly, most researches in herbal formulae were limited to find the evidence of herbal formulae's effects on some certain “diseases” [4–8].

 As we know, Chinese herbal formulae should aim to “ZHENG,” not to “disease.” Li et al. [9–11] had designed some systemic network method using public disease and drug component information to analyze the complexity of ZHENG and herbal formulae. For example, they had divided many diseases into cold ZHENG and hot ZHENG.

**Figure 1:**
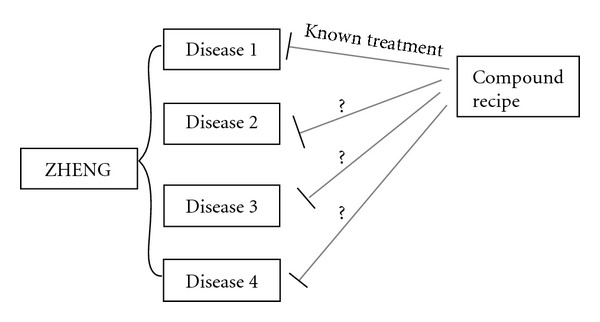
Prediction of herbal formulae's new treatment with the theory of “same ZHENG in different diseases.” Many herbal formulae, whose effect on a certain disease had been confirmed, might also treat other diseases with the same ZHENG.

 Since one ZHENG could relate many diseases and herbal formulae aimed to ZHENG, many herbal formulae, whose effect on a certain disease had been confirmed, might also treat other diseases with the same ZHENG (Figure 1).

In order to prove this idea, high-throughput gene microarrays were analyzed. The microarrays were collected from patients with QiXuXueYu ZHENG (Qi-deficiency and Blood-stasis syndrome) before treatment and treated with Fuzheng Huayu Capsule by a high-throughput drug similarity comparison method, we called it pathway-based similarity comparison (PBSC).

QiXuXueYu is a ZHENG whose patients suffer important energy deficiency and blood stasis. It is related with many different diseases such as diabetes mellitus [12, 13], dyslipidemia [14], hypertension [15], hepatitis, and liver cirrhosis [16]. This phenomenon is called “Same ZHENG in different diseases.” Fuzheng Huayu Capsule is a recipe on the basis of Chinese medicine theory in treating liver fibrosis [17] with QiXuXueYu ZHENG, but few researches had been done to find its treatment on other diseases above.

The PBSC method was based on a microarray database “Connectivity Map” (cMap) [18], which collect microarrays corresponding to treatment of 164 different small molecules in different human cell lines. In association with the cMap, a lot of groups explored its usage in various applications, including drug resistance analysis [19], and toxicity prediction [20], But no one used this data resource to predict new treatment of Chinese herbal formulae.

We first apply the cMap database consistent with high-throughput expression data to predict new treatment of Chinese herbal formulae. In our results, there were many drug molecules screened out, including antihyperglycemic, antihyperlipidemic, hypotensive, anti-inflammatory, and antifibrosis drugs and some molecules having global effects. By integrating all the molecules' information, a Fuzheng Huayu Capsule mechanism map was obtained and Fuzheng Huayu Capsule had both short-term treatment effect and long-term prevention and healthcare effect. Furthermore, clinical blood-tested data were used to verify our prediction and finding that Fuzheng Huayu Capsule can really relieve the patients suffering liver cirrhosis combined with diabetes mellitus or dyslipidemia.

## 2. Material and Methods

### 2.1. Samples

There were six blood samples, in which four samples were from two QiXuXueYu ZHENG patients (patients A and B) in both states of before treatment and being treated with Fuzheng Huayu Capsule (3200 mg ∗ 3 times/day, 24 weeks). The rest two samples were from QiXuXueYu ZHENG patients (patient C) in both states of before treatment and being treated with placebo (vehicle). All patients were suffering liver cirrhosis from Shanghai Longhua Hospital and had signed an agreement with us. The blood samples were morning fasting venous blood and saved in −20°C with 150 *μ*L EDTA.

 Except for the 6 samples, there were additional 360 blood samples from 180 QiXuXueYu ZHENG patients with in both states of before treatment and being treated with Fuzheng Huayu Capsule, and blood tests were taken from these samples to verify our prediction. All the 180 patients were suffering liver cirrhosis. But these samples were at first not collected to prove the effect of Fuzheng Huayu Capsule on hyperglycemia or dyslipidemia, so the samples of patients suffering liver cirrhosis combining hyperglycemia or dyslipidemia were not very abundant. Seventeen patients had higher fasting blood-glucose (GLU), 31 patients had higher postprandial blood sugar (PPG), and 21 patients had higher glycated hemoglobin (Hb1Ac). Fifteen patients' total cholesterols (T-ch) were abnormal. Among them, 7 patients had higher T-ch than the normal range, while 8 patients had lower T-ch than the normal range. Eighteen patients' Total triglycerides (TGs) were abnormal. Among them, 11 patients had higher T-ch than the normal range, while 7 patients had lower T-ch than the normal range.

### 2.2. RNA Extraction and Microarrays

 The TRIzol reagent (Invitrogen Life Technologies Company) was used to extract RNA of leukocyte from the whole blood of the 6 samples, then did a Quality Control with NanoDrop ND-1000. 

cDNA was obtained through the Invitrogen first-strand cDNA synthesis using M-MLV RT and added RNA polymerase to degrade RNA. cDNA labelling and hybridizations on NimbleGen Homo sapiens 12 × 135 K Array (Roche, CAT No. A6484-00-01) were performed according to the manufacturer's protocol.

### 2.3. Microarray Data Analysis

 Microarray data analysis was performed using the GenePix software. Raw expression data were log2-transformed and normalized by quantile normalization. Probes were considered robustly expressed if signal/noise ratio (SNR) < 2.

### 2.4. Connectivity Map (cMap) Database

 “Connectivity Map” is a reference collection of gene-expression profiles from cultured human cells treated with bioactive small molecules or drug molecules [18]. The data set was composed of mRNA expression data for 164 distinct small-molecules and corresponding vehicle controls applied to human cell lines. All these data were by means of Affymetrix GeneChip microarrays. We had downloaded total of 564 gene expression profiles, representing 453 individual instances at http://www.broad.mit.edu/cmap/.

### 2.5. Pathway Set

Gene sets were needed to sort out genes according to meaningful signal pathways. A set called Sigpathway [21] was used in our method. These gene sets are an integration of different pathway databases, including Biocarta, KEGG, BioCyc, pathway-specific microarray annotations, and >5,000 gene sets from Gene Ontology. The Sigpathway was available as an R package on http://www.bioconductor.org/packages/devel/bioc/html/sigPathway.html.

### 2.6. Pathway-Based Similarity Comparison (PBSC) Method

**Figure 2:**
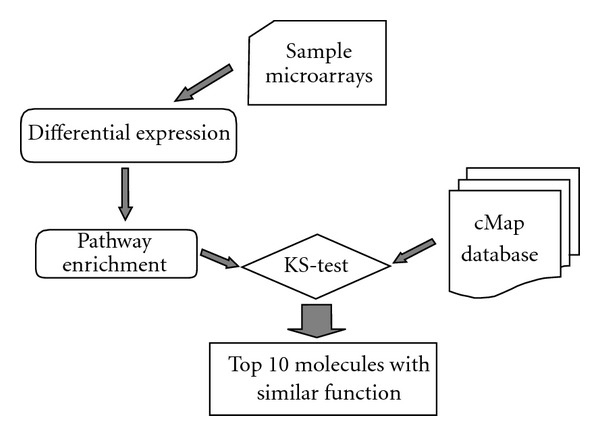
The process of PBSC method. 2-fold change was used as threshold for differential expression, and then Gene Set Enrichment Analysis (GSEA) was performed in every pathway. pathways, whose *P*-values obtained from GSEA was smaller than 0.05, were selected. Based on the selected Pathways, the expression pattern similarity between the microarrays of ours and in the cMap Database in every pathway was calculated using the KS-test.

 The process of PBSC was showed in Figure 2. At first 2-fold change was used as threshold for differential expression in every sample pair (treated with Fuzheng Huayu Capsule and before treatment), and then Gene Set Enrichment Analysis (GSEA) was performed in every pathway. Pathways, whose *P*-values obtained from GSEA was smaller than 0.05, were selected. Based on the selected pathways, the expression pattern similarity between the microarrays of ours and in the cMap Database in every pathway was calculated using the KS-test, which was recommended by Li et al. [22].

The progress of KS-test is as follows:

(1) p=Max⁡tj=1[jt−V(j)N],n=Max⁡tj=1[V(j)N−(j−1)t]KS={P,−n,  (P>n),(n>p).,

In the formula above, *t* is the number of genes in either the up- or down-regulated gene groups and *j* is the jth gene according to the rank of differential expression. *N* is the number of total genes in array, and the position of the *j*th gene in the rank ordered whole gene list is *V*(*j*). 

The result of similarity (KS value) in every pathway would be either positive or negative (“positive” displays the similar effects and “negative” displays the reversed effects). The top 10 reference chemicals which had the most similar pathway (both positive and negative) numbers were selected for each analysis.

All the process above was executed in R (Statistical software).

### 2.7. Statistic Analysis

 From the blood test data of patients suffering liver cirrhosis combining hyperglycemia or dyslipidemia, some indexes related with hyperglycemia or dyslipidemia were extracted, including fasting blood-glucose (GLU), postprandial plasma glucose (PPG), glycated hemoglobin (HbA1c), total cholesterol (T-ch) and total triglyceride (TG). The blood test data were expressed as means ± SD. Comparisons between before treatment and after treatment were performed by Student's *t*-test. The level of significance was set at *P* < 0.05. *t*-test was executed in R.

## 3. Results and Discussion

### 3.1. Differential Expression and Pathway Enrichment

The samples from three patients (patients A, B, and C) were, respectively, analyzed by PBSC method. The microarray data of patient B showed more difference expression genes (4375 up, 3066 down) than patient A (1642 up, 1743 down) between being treated with Fuzheng Huayu Capsule and before treatment. In other words, the recipe produced a greater effect on patient B.

Similarly, patient B showed more pathway changes than patient A in the pathway enrichment analysis (67 pathways versus 48 pathways). Many pathways were larger primary metabolic process; some smaller pathways were presented in Table 1. In the smaller pathways, the ubiquitin cycle with the protein catabolic metabolism seemed to be very important in our result. But so many larger primary metabolic processes can also contain, suggested that the effects of Fuzheng Huayu Capsule may be as a whole-regulated mechanism. 

Though patient C was treated with placebo, the microarray data also had many differential expression genes (2297 up, 1723 down). But these genes were in disorder and do not enrich many effects. Only 4 pathways were enriched (Table 1).

 There were many factors leading to the large difference before and after treatment even for placebo, such as the patients' situation and nursing care during the process of treatment. More repeated microarray examples with repeated experiment would be collected in future to improve the data unbalance.

### 3.2. The Top 10 Molecules Had Similar Gene Expression Pattern and with Fuzheng Huayu Capsule

 After pathway enrichment analysis, the similarity search for every pathway between the microarray data and cMap Database was executed. For each patient, top 10 drug molecules in cMap Database sharing the largest number of significantly affected pathway numbers with Fuzheng Huayu Capsule (patients A and B) or placebo (patient C) were presented in Table 2. “+” indicates the number of pathways positively correlated; “−” indicate the number of pathways negatively correlated.

**Figure 3:**
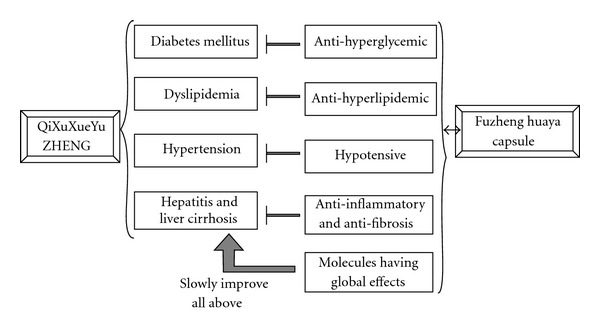
Predicted effects of Fuzheng Huayu Capsule. There were many drug molecules predicted by our method can reflect one part of effects of the formulae, including anti-hyperglycemic (chlorpropamide, metformin), anti-hyperlipidemic (pirinixic acid), hypotensor (verapamil, dexverapamil), anti-inflammatory, and anti-fibrosis drugs (tacrolimus, sirolimus, and mesalazine), molecules having global effects (estrogen, genistein).

Almost all drug molecules presented in Tables 2(a) and 2(B) had positive pathways, so these molecules had similar gene expression pattern and effects with Fuzheng Huayu Capsule in such pathways. These drug molecules could be classified by their effects, including anti-hyperglycemic (Chlorpropamide, Metformin), anti-hyperlipidemic (Pirinixic acid), hypotensor (Verapamil, Dexverapamil), anti-inflammatory and Anti-fibrosis drugs (Tacrolimus, Sirolimus, Mesalazine) and some molecules having global effects (Estrogen, Genistein). The new effects of Fuzheng Huayu Capsule was predicted and summarized in Figure 3.

In anti-inflammatory and Anti-fibrosis drugs, Tacrolimus in Patient A and Sirolimus in Patient B were immunosuppressant drugs. Tacrolimus was a calcineurin inhibitor. Sirolimus inhibits the response to IL-2, and thereby blocks activation of T- and B-cells. They can also ameliorate fibrosis [23, 24]. Mesalazine was also an anti-inflammatory drug [25]. These results showed the known effects of Fuzheng Huayu Capsule.

In Anti-hyperglycemic, Chlorpropamide was the only molecule positive in both patients A and B. It was a drug in the sulphonylurea class used to treat type 2 diabetes mellitus [26]. Sulfonylureas bind to K^+^ channel on the cell membrane of pancreatic beta cells, Then depolarization opens voltage-gated Ca^2+^ channels. The rise in intracellular calcium leads to increased fusion of insulin granulae with the cell membrane, and therefore increased secretion of (pro)insulin [26]. Metformin was also a drug used to treat type 2 diabetes mellitus [27].

In hypotensive, verapamil and dexverapamil were calcium channel blockers of the phenylalkylamine class. It had been used in the treatment of hypertension [28]. Calcium channels were present in the smooth muscle that lines blood vessels. By relaxing the tone of this smooth muscle, calcium-channel blockers dilate the blood vessels [28].

In anti-hyperlipidemic, pirinixic acid was a hypolipidemic, peroxisome proliferator-activated receptor [29]. There was a special situation about pirinixic acid in our result. pirinixic acid in patient A was negative to Fuzheng Huayu Capsule, while it was positive in patient B. This means Fuzheng Huayu Capsule could play a role like pirinixic acid to reduce blood lipids and play a reversed role to raise blood lipids. Some other researches had found the bidirectional regulation effect of TCM [30, 31]; it was an unique feature of TCM which was rare in western medicine. We also did some verification on the bidirectional regulation effect of Fuzheng Huayu Capsule in Section 3.4.

In molecules having global effects, genistein was one of several known isoflavones found in leguminous plants, causing effects in the body similar to those caused by the hormone estrogen (estradiol). Isoflavones and estradiol can regulate blood glucose [32], blood fat [33], blood pressure [34], inflammation [35] with many long-term systemic effect. 

There were also some molecules in Tables 2(a) and 2(b) that did not have many relationships with the above diseases (diabetes mellitus, dyslipidemia, hypertension, hepatitis and liver cirrhosis). Tetraethylenepentamine was negative to Fuzheng Huayu Capsule in patient B and it was a harmful substance to people, so this result hinted that tetraethylenepentamine would aggravate the illness of patients. Chlorpromazine in Patient B and trifluoperazine in patient A were typical antipsychotic. Exisulind and novobiocin were drugs used to treat cancer. But in Table 2(c), the placebo also shows these effects by some molecules, though the number of pathways was very small. Haloperidol, clozapine, fluphenazine, and prochlorperazine were all antipsychotic. Trichostatin A was an anti-tumor agent. So antipsychotic and anti-tumor were not the main effects of Fuzheng Huayu Capsule. This effect might have some other cause. There might be some bias in examples or the patients may had some comfort mentality after treatment and then show some effects of psychotropic drugs.

### 3.3. The Potential Multiple-Target Molecular Pharmacology of Fuzheng Huayu Capsule

**Figure 4:**
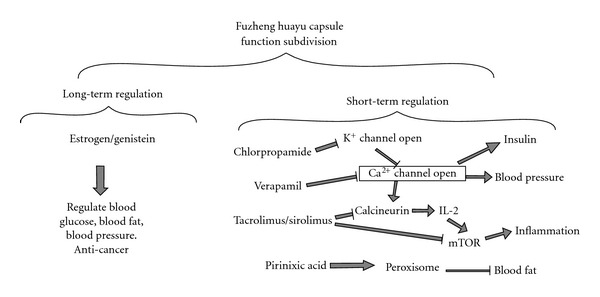
potential multiple-target molecular pharmacology of Fuzheng Huayu Capsule. Genistein and estradiol were assigned to long-term regulation group and other drugs were assigned to short-term regulation group. Ca^2+^ related effects might have core effects in the molecular pharmacology of the short-term group.

 Integrating all the information above, a mechanism map of Fuzheng Huayu Capsule effects was built up as follows (Figure 4). The drugs in our results were divided into two big groups, long-term regulation group and short-term regulation group. Genistein and estradiol were assigned to long-term regulation group, because they had many sustained effects on our health and we can get them by daily diet or produce them by ourselves.

Chlorpropamide/metformin, tacrolimus/sirolimus, verapamil/dexverapamil, and Pirinixic acid were all assigned to short-term regulation group. The Ca^2+^ related effects had a core effects in the molecular pharmacology of the short-term effects of Fuzheng Huayu Capsule. Ca^2+^ is an important second messenger in many cell primary metabolic processes such as inflammation, metabolism, apoptosis, smooth muscle contraction, intracellular movement, nerve growth, and the immune response.

 There was an important point that these small molecules were selected by effects, not by compound structure. The PBSC method could find molecules having similar effects, not similar structure. In fact, many molecules in our result had considerable side effect, but Fuzheng Huayu Capsule do not have considerable side effect.

Therefore, our result did not means there were some molecules in Fuzheng Huayu Capsule having similar structure or drug target with the molecules in our result. They should only have similar effect on downstream mechanism, such as Ca^2+^ related pathway.

### 3.4. Blood Test Verification

 To verify our prediction, we took use of some existing data of blood tests. The data included 360 samples from 180 QiXuXueYu ZHENG patients in both states of before treatment and being treated with Fuzheng Huayu Capsule. But these samples were at first not collected to prove the effect of Fuzheng Huayu Capsule on hyperglycemia or dyslipidemia. So only a part of the patients were suffering hyperglycemia or dyslipidemia, while all the 180 patients were suffering liver cirrhosis. The laboratory values of blood glucose and blood lipid in the data of blood tests were showed in Table 3.

 In blood glucose tests, 17 patients had higher GLU before treatment and 7 patients (41%) got back to normal range after treatment with Fuzheng Huayu Capsule. According to the treatment, total average GLU of the 17 patients went down from 7.42 to 6.52, and 12 of 31 patients (38%) got back to normal range. PPG and the total average PPG went down from 10.84 to 8.72. Moreover, 16 of 21 patients (76%) got back to normal range of HbA1c and the total average HbA1c went down from 7.48 to 5.86. There were the significant difference in data of PPG and HbA1c between before and after treatment (*P* < 0.05).

 In blood lipid tests, 7 patients had higher T-ch than the normal range, while 8 patients had lower T-ch than the normal range, and 11 patients had higher TG than the normal range, while 7 patients had lower T-ch than the normal range. The average values of all sets of patients tended to normal after treatment. May be it was lack of samples, the data between before and after treatment did not have significant difference except that lower T-ch went up. Interestly, not only the higher T-ch and TG were down regulated, but also the lower T-ch and TG were up regulated by Fuzheng Huayu Capsule, which may be a characteristic of herbal formulae with multi-compounds.

Previous study also reported that Fuzheng Huayu had comprehensive effect on patients suffering liver fibrosis along with Diabetes mellitus [36]. These results suggested that Fuzheng Huayu Capsule could really relieve the patients suffering liver cirrhosis combined with diabetes mellitus and might have biphasic regulation effects on dyslipidemia.

Scince the research was to mainly explore a method to predict new effects of Fuzheng Huayu Capsule through integrat the information of ZHENG, herbal formula, and diseases, the experimental examples were not very abundant. We would carry out studies on large samples in future.

## 4. Conclusion

We introduced a high-throughput gene microarrays-based method (PBSC) to predict the potential effects of Fuzheng Huayu Capsule, a Chinese herbal formula on liver cirrhosis with QiXuXueYu ZHENG. The predicted results showed that the comprehensive effects of Fuzheng Huayu Capsule might be including Anti-hyperglycemic, anti-hyperlipidemic, hypotensive and anti-inflammatory, and Anti-fibrosis drugs. To verify our prediction, we had also taken the blood tests and got the effectiveness of Fuzheng Huayu Capsule on liver cirrhosis combined with diabetes mellitus or dyslipidemia. Further researches must get more samples to confirm the potential effects of Fuzheng Huayu Capsule.

 Our research results suggested that the PBSC method is effective to find small molecules which had similar gene expression patterns and effects with herbal formulae and offer invaluable information for predicting new treatment application of herbal formulae.

## Figures and Tables

**Table 1:** Partial pathway enrichment.

Patient A pathways	Patient B pathways	Patient C pathways
Ubiquitin cycle	Uubiquitin cycle	Cellular protein metabolic process
Leukocyte migration	Apoptosis	Protein metabolic process
Transmembrane receptor protein tyrosine kinase signaling pathway	Ubiquitin-dependent protein catabolic process	Cellular macromolecule metabolic process
Nitrogen compound metabolic process	Regulation of actin polymerization and/or depolymerization	rRNA metabolic process
Regulation of angiogenesis	Nucleocytoplasmic transport	

**Table:** (a) Patient A

cMap ID	Drug molecule	Dose	Pathway counts
169	Tacrolimus	1 uM	22+
383	Cobalt chloride	100 uM	21+
144	Chlorpropamide	100 uM	20+ 1−
641	Benserazide	10 uM	20+
576	Novobiocin	100 uM	20−
487	Pirinixic acid	100 uM	20−
421	Trifluoperazine	10 uM	20+
314	Exisulind	50 uM	20+
284	Tacrolimus	1 uM	20+
268	Genistein	1 uM	20+

**Table:** (b) Patient B

cMap ID	Drug molecule	Dose	Pathway counts
487	Pirinixic acid	100 uM	53+
161	Verapamil	10 uM	52+
2	Metformin	10 uM	52+
419	Chlorpromazine	10 uM	49+
49+	Sirolimus	100 nM	
49+	Dexverapamil	10 uM	
141	Chlorpropamide	100 uM	49+
122	Alpha-estradiol	10 nM	49+
457	Tetraethylenepentamine	100 uM	47−
124	Mesalazine	100 uM	46+

**Table:** (c) Patient C

cMap ID	Drug molecule	Dose	Pathway counts
608	NU-1025	100 uM	4−
418	Haloperidol	10 uM	4+
282	Fludrocortisone	1 uM	4+
1072	Trichostatin A	1 uM	3+
984	Acetylsalicylic acid	100 uM	3+
1009	Clozapine	10 uM	3+
1017	Fluphenazine	10 uM	3+
1024	Haloperidol	10 uM	3+
995	Prochlorperazine	10 uM	3−
887	Celastrol	3 uM	3+

**Table 3:** The laboratory parameters of blood glucose and blood lipid.

Laboratory parameters	Total patient number	Improved patient number	Normal value range	Total average before treatment	Total average after treatment	*P* value (*T* test)
GLU (mmol/L)	17	7	3.89–6.1	7.42	6.52	0.068
PPG (mmol/L)	31	12	3.9–7.8	10.84	8.72	0.025
HbA1c (%)	21	16	4.3–6.5	7.48	5.86	0.00002
T-ch (higher) (mmol/L)	7	4	2.86–5.98	7.19	6.49	0.383
T-ch (lower) (mmol/L)	8	7	2.86–5.98	2.66	3.76	0.0207
TG(higher) (mmol/L)	11	7	0.58–1.88	2.61	1.93	0.105
TG (lower) (mmol/L)	7	5	0.58–1.88	0.52	0.94	0.106
